# SLA2 is Associated With Immune evasion and Exhaustion of CD8 ^+^ T Cells in Gastric Cancer

**DOI:** 10.1111/jcmm.71164

**Published:** 2026-05-10

**Authors:** Yujia Zhang, Xiaoyan Li, Rongchao Xiang

**Affiliations:** ^1^ Department of Gastrointestinal Surgery Deyang People's Hospital Deyang China; ^2^ Finance Department Deyang People's Hospital Deyang China

**Keywords:** CD8^+^ T cells, gastric cancer, immune evasion, SLA2, tumour microenvironment

## Abstract

The Src‐like adaptor 2 (SLA2) functions as a negative regulator of T cell receptor signalling. However, its involvement in the tumour microenvironment (TME) of gastric cancer (GC) remains unexplored. In this study, we found that SLA2 expression was significantly elevated in GC tissues, and a high level of SLA2 was associated with poor prognosis in GC patients. Bioinformatics analyses revealed a close association between SLA2 and TME in GC. Single‐cell RNA sequencing analysis indicated that SLA2 was significantly enriched in CD8^+^ T cells in GC tissues. Functional validation demonstrated that SLA2 overexpression contributed to the exhaustion of CD8^+^ T cells by suppressing their proliferation, upregulating the expression of exhaustion markers, reducing the secretion of effector cytokines (IFN‐γ and TNF‐α) and impairing cytotoxic function. SLA2 knockdown in in vitro‐generated exhausted CD8 T cells significantly alleviated T cell exhaustion. Mechanistically, we found that inverse promoter methylation and active histone marks (H3K27ac, H3K4me3 and H3K4me1) may regulate SLA2 expression. Our findings suggest that SLA2 may modulate the TME and promote immune evasion via CD8^+^ T cell exhaustion in GC.

**Abbreviations**
CNA: copy number alteration
CNVs: copy number variations
GC: gastric cancer
ICIs: Immune checkpoint inhibitors
MDSCs: myeloid‐derived suppressor cells
MHC: major histocompatibility complex
NK: natural killer
PD‐1: programmed death protein 1
SLA2: Src‐like adaptor 2
TAMs: tumour‐associated macrophages
TME: tumour microenvironment

## 1 Introduction

Gastric cancer (GC) ranks as the fifth most common malignant neoplasm and is the fifth leading cause of cancer‐related death [1]. It is estimated that 968,350 new GC cases and 659,853 GC‐related deaths occurred worldwide in 2022. Moreover, the incidence and mortality rates of GC are 1.84‐fold higher in men than in women. According to recent analyses of malignant tumour prevalence in China, GC ranks fifth in terms of incidence among all malignant tumours and third among digestive tract cancers [2]. Despite significant advances in treatment strategies, including chemotherapy, radiotherapy, immunotherapy and surgical resection, the cure rate for GC remains suboptimal. The five‐year survival rate for patients with GC remains unfavourable due to local recurrence and distant metastasis [3]. Thus, gaining a more comprehensive insight into the mechanisms underlying gastric cancer progression and identifying novel therapeutic targets are essential for improving treatment outcomes and reducing mortality rates.

Immune checkpoint inhibitors (ICIs), particularly those targeting programmed death protein 1 (PD‐1) and its ligand PD‐L1, have become the leading immunotherapeutic approach for treating GC [4, 5]. Within the intricate tumour microenvironment (TME) of GC, persistent antigen exposure and chronic inflammatory signals promote the functional exhaustion of tumour‐infiltrating CD8^+^ T cells [6, 7, 8]. These exhausted cells exhibit markedly impaired effector functions, including reduced cytokine production and cytotoxic activity, while maintaining high expression levels of multiple inhibitory receptors such as PD‐1, TIM‐3 and LAG‐3 [9, 10]. This sustained upregulation establishes a robust ‘immune checkpoint’ barrier, allowing tumour cells to escape immune surveillance and elimination. Consequently, CD8^+^ T cell exhaustion is widely recognized as a central mechanism underlying immune evasion and therapeutic resistance in GC [11, 12]. Comprehensive investigation into the molecular mechanisms governing CD8^+^ T cell exhaustion, alongside the development of immunotherapeutic approaches aimed at reversing this dysfunctional state and restoring T cell functionality, has emerged as a central focus and rapidly advancing area in contemporary GC research.

The Src‐like adaptor 2 gene (SLA2, also known as SLAP‐2) is one of the Src‐like adaptor proteins whose main function is to inhibit the activation of the activated T cell nuclear factor induced by the T cell antigen receptor [13]. SLA2 serves as a prognostic indicator in patients with early‐stage pancreatic ductal adenocarcinoma following pancreaticoduodenectomy [14] and breast cancer [15]. Additionally, SLA2 is significantly associated with immune response function in non‐small cell lung cancer [16] and associated with immune cell infiltration within the TME in neck squamous cell carcinoma [17]. However, a significant knowledge gap remains in the context of GC. Systematic investigations into the dysregulation of SLA2 expression in GC tissues, its correlation with clinicopathological features and patient prognosis, and its precise distribution and function within immune cell subsets of the TME are currently lacking. Furthermore, while the role of SLA2 in dampening TCR signalling conceptually implicates it in T cell exhaustion, direct evidence linking SLA2 to CD8^+^ T cell dysfunction in GC has not been established. Bridging this gap is critical for identifying novel mechanisms of immune evasion and potential therapeutic targets in gastric cancer.

In this study, we comprehensively analysed the expression of SLA2 and its association with the prognosis, immune infiltrations and immune evasion in GC. Our results demonstrate that SLA2 modulates the TME and promotes immune evasion via CD8^+^ T cell exhaustion in GC.

## 2 Materials and Methods

### 2.1 Patients and Samples

All procedures were conducted in compliance with applicable guidelines and regulations and were approved by the Medical Ethics Committee of Deyang People's Hospital. A total of 18 gastric cancer tissue samples and corresponding adjacent non‐tumour tissues were collected before treatment. Written informed consent was obtained from all participants involved in the study.

### 2.2 Immunofluorescence Assay

Paraffin‐embedded tissue sections were processed for immunofluorescence staining. Tissue samples were incubated overnight with a rabbit recombinant monoclonal anti‐SLA2 antibody (Thermo Fisher Scientific), followed by incubation with a goat anti‐rabbit IgG secondary antibody conjugated to Alexa Fluor 594 (ab150080, Abcam) in the dark at room temperature. Nuclei were visualized using DAPI staining (Beyotime, C1006), and fluorescence signals were observed under a confocal laser scanning microscope.

### 2.3 The University of ALabama at Birmingham CANcer Data Analysis Portal (UALCAN)

The UALCAN platform (http://ualcan.path.uab.edu/) was utilized to analyse SLA2 expression levels and their correlation with clinicopathological features, including cancer stage and tumour grade, in stomach adenocarcinoma (STAD).

### 2.4 The Human Protein Atlas (HPA)

The HPA platform (https://www.proteinatlas.org/) was employed to examine SLA2 expression across diverse cell lines (including common models and multiple immune cell types).

### 2.5 Gene Expression Profiling Interactive Analysis (GEPIA)

GEPIA (http://gepia.cancer‐pku.cn/index.html) is an online resource designed for gene expression profiling using data from The Cancer Genome Atlas (TCGA) and GTEx databases. In this study, the tool was utilized to assess SLA2 expression levels in both TCGA‐STAD and GTEx datasets.

### 2.6 Kaplan–Meier Plotter Database Analysis

The KM Plotter (http://kmplot.com) was employed to evaluate the prognostic significance of SLA2 in gastric cancer. Patients were stratified into two groups based on gene expression levels to assess overall survival (OS), progression‐free survival (PFS) and post‐progression survival (PPS), with differences determined using log‐rank tests and corresponding *p*‐values.

### 2.7 Tumour Immune Estimation Resource (TIMER) Database

The TIMER database (https://compbio.cn/timer3/) was employed to compare SLA2 expression levels in tumour tissues with those in adjacent normal tissues. Using the Immune module, we investigated the association between SLA2 expression and the infiltration levels of various immune cell types, as well as its correlation with the TIDE (Tumour Immune Dysfunction and Exclusion) score and ESTIMATE score. The Cancer Exploration module was used to assess the correlation between SLA2 and the expression levels of other genes in STAD. To explore the correlation between SLA2 and immunotherapy response in GC. RNA‐seq profiles from the European Nucleotide Archive (ENA) database (ERP107734, 45 patients with advanced GC receiving pembrolizumab monotherapy) [18] were processed using TIMER3. Treatment responses were categorized according to the RECIST criteria as complete response (CR), partial response (PR), stable disease (SD), or progressive disease (PD). SLA2 expression was compared across clinical response groups: responders (CR or PR) versus non‐responders (SD or PD). We further evaluated the predictive capacity of SLA2 expression for anti‐PD‐1 treatment response in gastric cancer patients by computing receiver operating characteristic (ROC) curves using TIMER3.

### 2.8 SLA2 Mutation and Methylation Analysis

The copy number alterations (CNA) and mutational landscape of SLA2 in GC were analysed using the web‐based platform cBioPortal (http://www.cbioportal.org). The integration and visualization of SLA2 expression, DNA methylation levels and the exact genomic positions of CpG sites were performed through the online tool MEXPRESS (https://mexpress.be/). Additionally, the Shiny Methylation Analysis Resource Tool (SMART, http://www.bioinfo‐zs.com/smartapp/) was employed to examine SLA2 methylation patterns in STAD and to assess the association between CpG island methylation and SLA2 expression levels.

### 2.9 Correlation Analysis Between SLA2 and Immunoregulators

The association between SLA2 and lymphocytes, immunostimulatory molecules, immune checkpoint inhibitors, MHC molecules, receptors and chemokines across multiple cancer types was explored using the TISIDB database (http://cis.hku.hk/TISIDB/).

### 2.10 Single‐Cell Sequencing Analysis

The Tumour Immune Single Cell Hub (TISCH) is a curated RNA‐sequencing database that enables in‐depth analysis of the TME across multiple cancer types by providing cell‐type‐specific annotations. In this study, TISCH was utilized to explore SLA2 expression profiles across various cell populations in multiple single‐cell datasets related to STAD.

### 2.11 Cell Culture

Human AGS and MKN‐45 cells were acquired from Procell (Wuhan, China) and authenticated via short tandem repeat (STR) profiling. The cells were maintained in RPMI 1640 medium containing 10% foetal bovine serum (FBS) and 1% penicillin‐streptomycin (Gibco), under standard culture conditions of 37°C in a humidified atmosphere with 5% CO_2_.

### 2.12 In Vitro T Cell Exhaustion

Peripheral CD8^+^ T cells were isolated from PBMCs of healthy individuals using Lymphoprep density gradient centrifugation (Stemcell Technologies), followed by positive selection with the EasySep Human CD8^+^ T Cell Isolation Kit (Stemcell Technologies). Purified CD8^+^ T cells were activated using ImmunoCult Human aCD3/aCD28 T Cell Activator (Stem Cell Technologies) and initially cultured in OpTmizer T Cell Expansion Medium (Gibco), supplemented with recombinant human IL‐2 (10 ng/mL) for 48 h. Following the initial stimulation, the singly stimulated control group was continuously cultured in complete media. Moreover, T cell exhaustion was induced through repeated antigenic stimulation—administered every 48 h for six consecutive cycles [19]. Exhaustion was confirmed by elevated PDCD1, TIGIT, HAVCR2 and LAG3 expression and reduced IFN‐γ/TNF‐α secretion.

### 2.13 Transfection

Lentiviral vectors carrying SLA2 overexpression plasmids were constructed and packaged by GenePharma (Shanghai, China) for cellular transduction. Small hairpin RNAs (shRNAs) targeting SLA2, KMT2C, KMT2D, EP300, or KAT2B, along with a non‐targeting scrambled control (shNC), were synthesized by GenePharma. Each shRNA sequence was cloned into the pLKO.1 vector. Lentiviral particles were generated by transfecting packaging cells with the respective recombinant plasmids; viral supernatants were collected 48 h after transfection and concentrated by ultracentrifugation. Activated CD8^+^ T cells were transduced with the lentiviral particles at a multiplicity of infection (MOI) of 10 over a 48‐h period, followed by assessment of SLA2 expression levels to determine the efficiency of transduction.

### 2.14 Cell Proliferation Assay

Activated CD8^+^ T cells (2 × 10^4^ cells per well) were seeded into 96‐well plates and transduced with lentiviral vectors for 48 h. Subsequently, 10 μL of CCK‐8 solution was added to each well, and the plates were incubated at 37°C for 2 h. The absorbance was measured at 450 nm.

### 2.15 Quantitative Real‐Time PCR

Total RNA was extracted from clinical tissue samples and cultured cells using TRIZOL reagent (Invitrogen), followed by reverse transcription into cDNA using the PrimeScript RT Reagent Kit (Takara). Quantitative real‐time PCR was carried out with the SYBR Premix Ex Taq II Kit (Takara), employing β‐actin as an internal control. The relative expression of target genes was calculated by normalizing to β‐actin levels using the 2^−ΔΔCT^ method.

### 2.16 Enzyme‐Linked Immunosorbent Assay (ELISA)

The concentrations of IFN‐γ and TNF‐α in cell culture supernatants were measured using Human IFN‐γ ELISA Kit (PI521, Beyotime, Shanghai, China) and Human TNF‐α ELISA Kit (PT518, Beyotime), respectively, following the manufacturer's provided protocols.

### 2.17 Lactate Dehydrogenase (LDH)‐Based Cytolysis Assay

AGS or MKN‐45 cells were seeded into 96‐well plates in triplicate and allowed to adhere for 6 h. Subsequently, pre‐transfected CD8^+^ T cells were added at an effector‐to‐target (E: T) ratio of 5:1 for 12 h. Cytolytic activity was measured using a cytotoxicity LDH assay kit (GlpBio). The specific lysis percentage of tumour cells was determined by normalizing the LDH release to the maximum release in each group, with correction for background LDH levels from AGS cells cultured alone.

### 2.18 Chromatin Immunoprecipitation (ChIP)‐PCR

ChIP assays were carried out using the Pierce Magnetic ChIP Kit (Thermo Fisher Scientific). Briefly, the cells were fixed with 1% formaldehyde, and the cross‐linking reaction was terminated by adding glycine. Cells were then collected, lysed and sonicated on ice to shear the chromatin. Immunoprecipitation was performed using antibodies against Histone H3 mono‐methylated at Lys4 (ab8895, Abcam), Histone H3 tri‐methylated at Lys4 (ab8580, Abcam) and Histone H3 acetylated at Lys27 (ab4729, Abcam). Rabbit IgG was used as a negative control antibody. A 10% aliquot of the sheared chromatin before immunoprecipitation was retained as an input control. The enriched DNA fragments were subsequently analysed by quantitative PCR.

### 2.19 Statistical Analysis

Data analysis was conducted using GraphPad Prism 7 (San Diego, California, USA). For each experiment, three independent biological replicates were conducted. Data are presented as mean ± standard deviation (SD). The differences between the two groups were assessed using Student's *t*‐test. For comparisons among three or more groups, one‐way ANOVA followed by Tukey's post hoc test was employed. Survival curves were generated using the Kaplan–Meier method and compared by the log‐rank test. A *p*‐value less than 0.05 was considered statistically significant.

## 3 Results

### 3.1 SLA2 Is Increased in GC

**FIGURE 1:**
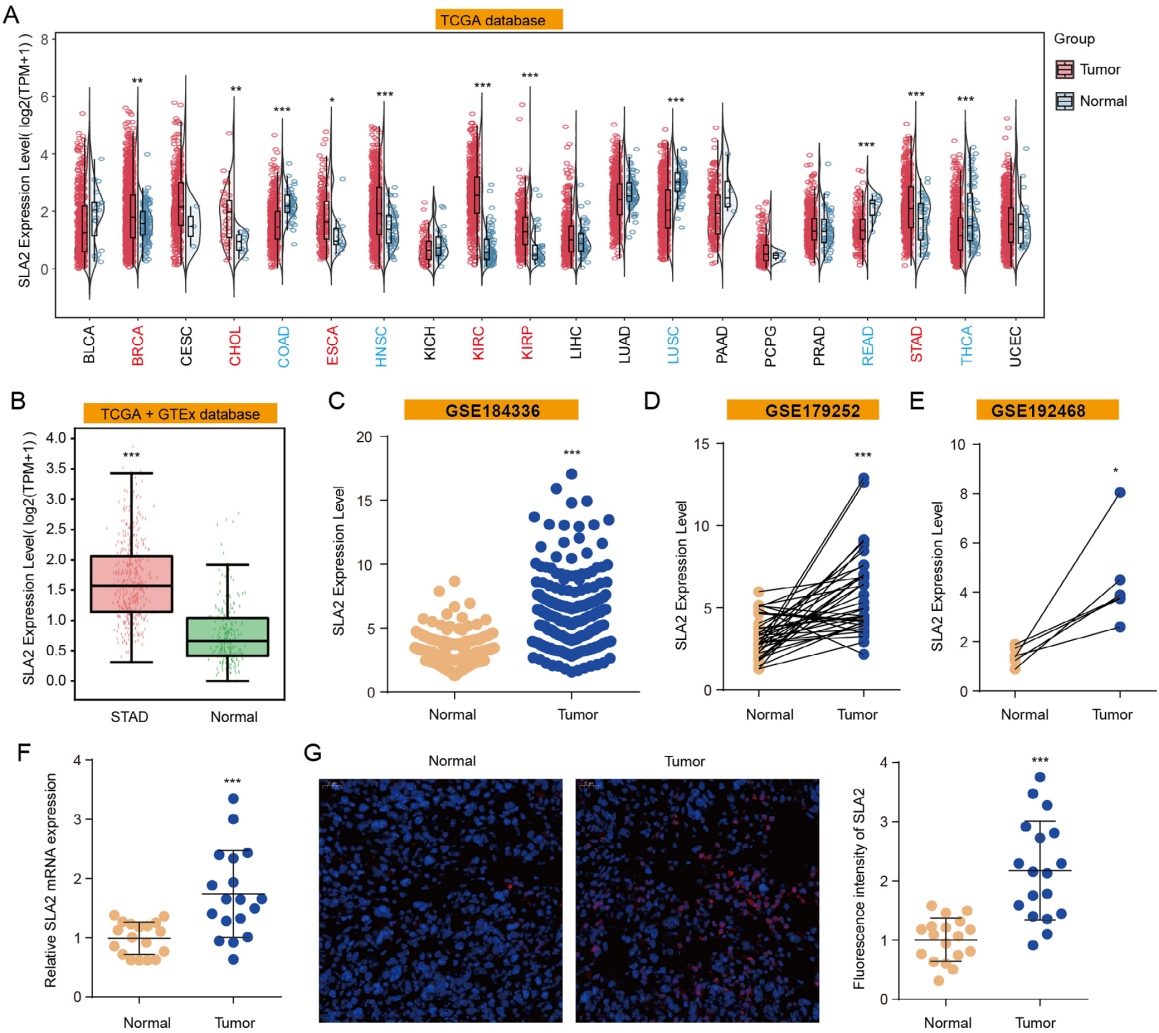
*SLA2 expression in GC patients. (A) Analysis of SLA2 expression across various cancer types using TIMER3.0*. The statistical significance computed by differential analysis (edgeR) on RNA‐Seq raw counts: *p < 0.05, **p < 0.01, ***p < 0.001. (B) Comparison of SLA2 expression in TCGA‐STAD samples combined with GTEx normal tissues via GEPIA3. Statistical significance was assessed by unpaired Student's t‐test: ***p < 0.001. (C) Analysis of SLA2 expression in GC and normal tissues in GSE184336 cohort. Statistical significance was assessed by unpaired Student's t‐test: ***p < 0.001. (D) Analysis of SLA2 expression in 38 paired GC and adjacent non‐tumour tissues in GSE179252 cohort. Statistical significance was assessed by paired Student's t‐test: ***p < 0.001. (E) Analysis of SLA2 expression in six paired GC and adjacent non‐tumour tissues in GSE192468 cohort. Statistical significance was assessed by paired Student's t‐test: *p < 0.05. (F) Assessment of SLA2 mRNA levels in a locally recruited cohort of gastric cancer patients (*n* = 18). Statistical significance was assessed by paired Student's t‐test: ***p < 0.001. (G) Immunofluorescence analysis of SLA2 protein expression in GC and adjacent normal tissues (*n* = 18). Statistical significance was assessed by paired Student's t‐test: ***p < 0.001.

To elucidate the specific role of SLA2 in GC, we systematically assessed its expression profile across various cancer types using the TIMER3.0 platform based on TCGA‐STAD data. Our analysis demonstrated that SLA2 expression was significantly upregulated in BRCA, CHOL, ESCA, KIRC, KIRP and STAD, whereas it was downregulated in COAD, HNSC, LUSC, PRAD and THCA (Figure 1A). Due to the limited availability of normal gastric tissue samples in TCGA, we integrated data from the GTEx database via GEPIA3 to validate SLA2 expression in STAD, which robustly confirmed its overexpression (Figure 1B). This finding was further corroborated by an independent analysis of the GSE184336 dataset, which also revealed elevated SLA2 levels in GC (Figure 1C). Importantly, paired‐sample comparisons from both GSE179252 and GSE192468 consistently showed significantly higher SLA2 expression in tumour tissues than in matched adjacent non‐tumour tissues (Figure 1D,E). To strengthen these bioinformatic findings, we experimentally validated SLA2 expression in a locally collected cohort of GC patients, confirming markedly increased mRNA levels in tumour specimens compared to adjacent normal tissues (Figure 1F). Immunofluorescence staining provided additional protein‐level evidence, showing pronounced upregulation of SLA2 in GC tissues (Figure 1G). Taken together, these multi‐source data consistently demonstrate that SLA2 is overexpressed in GC, suggesting its potential involvement in the progression of GC.

### 3.2 Association Between SLA2 Expression and Clinicopathological Features and Patient Outcomes

**FIGURE 2:**
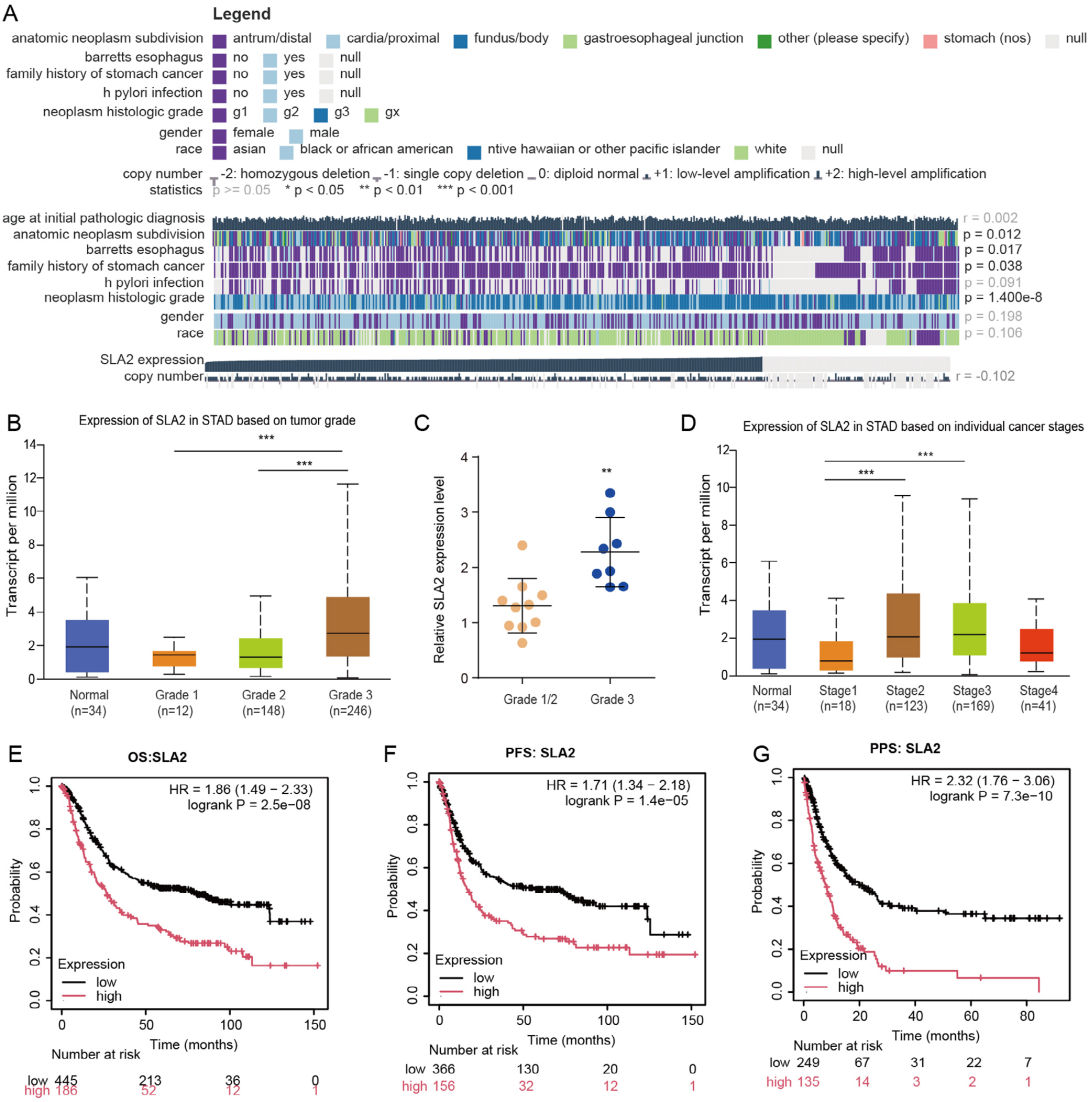
*Correlation between SLA2 expression and clinicopathological parameters and outcomes in GC*. (A) Assessment of the association between SLA2 expression and clinicopathological features using TCGA‐STAD data through the MEXPRESS platform. (B) Differential expression of SLA2 across tumour grades in STAD. Statistical significance was assessed by one‐way ANOVA followed by Tukey's post hoc test for multiple comparisons: ***p < 0.001. (C) Validation of SLA2 expression patterns according to tumour grade in an independent institutional cohort. Statistical significance was assessed by unpaired Student's t‐test: **p < 0.01. (D) Variation in SLA2 expression levels among distinct pathological stages in STAD. Statistical significance was assessed by one‐way ANOVA followed by Tukey's post hoc test for multiple comparisons: ***p < 0.001. (E–G) Kaplan–Meier survival analysis comparing OS, PFS and PPS between patient groups stratified by low versus high SLA2 expression. Statistical significance was assessed by the log‐rank test.

The relationship between SLA2 expression and clinicopathological characteristics was investigated using transcriptomic and clinical data from TCGA‐STAD. Analysis using the MEXPRESS platform revealed that SLA2 expression was significantly associated with key clinical variables, including anatomic neoplasm subdivision, barrett's oesophagus, family history of gastric cancer and tumour histologic grade (Figure 2A). Further evaluation using the UALCAN database demonstrated that SLA2 expression was higher in grade 3 tumours than in grades 1 and 2, indicating a strong association with higher tumour differentiation status in STAD (Figure 2B). This observation was validated in our institutional cohort, where high‐grade tumours consistently exhibited increased SLA2 expression relative to lower‐grade counterparts (Figure 2C). Additionally, the expression level of SLA2 in STAD was significantly associated with clinical stage progression (stage 1 to stage 4) (Figure 2D). SLA2 expression was markedly elevated in stage 2 and stage 3 tumours compared with stage 1. To assess its prognostic value, survival analyses were conducted using the KM Plotter–Gastric Cancer database. High SLA2 expression was significantly correlated with reduced overall survival (OS), progression‐free survival (PFS) and post‐progression survival (PPS) in GC patients (Figure 2E–G). Taken together, these results indicate that upregulation of SLA2 is closely linked to aggressive disease features and poor clinical outcomes in GC.

### 3.3 Association of SLA2 Expression With TME and Immune Evasion

**FIGURE 3:**
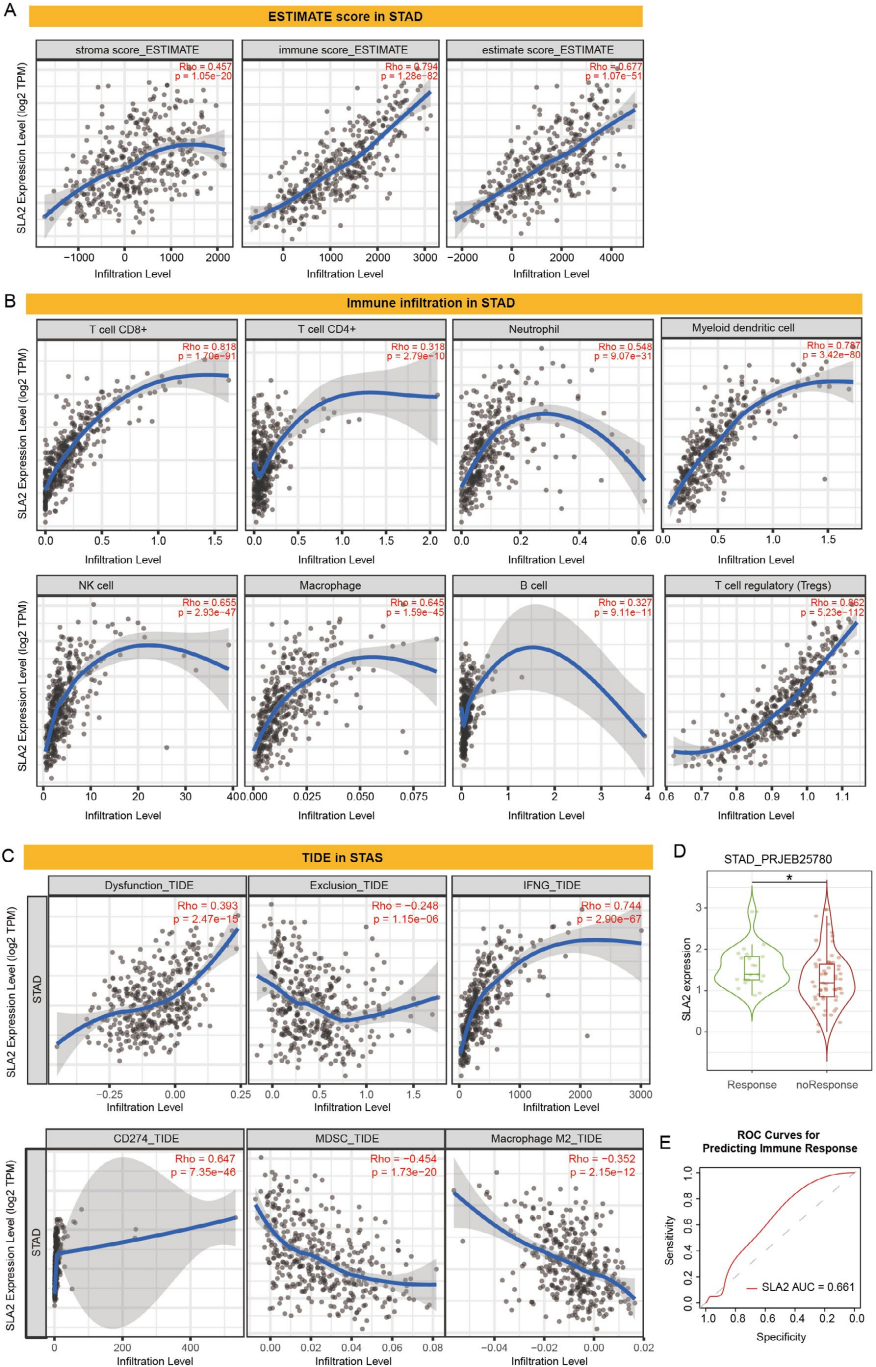
*Association of SLA2 with TME and immunotherapy response in STAD*. (A) Correlation between SLA2 expression levels and stromal score, immune score and ESTIMATE score in STAD. (B) Correlation between SLA2 expression and the abundance of various immune cell populations in the STAD tumour microenvironment. (C) Association between SLA2 expression and TIDE scores. (D) SLA2 expression levels in STAD patients stratified according to anti‐PD‐1 immunotherapy response outcomes based on the STAD_PRJEB25780 cohort. Statistical significance was assessed by unpaired Student's t‐test: *p < 0.05. (E) ROC curves assessing the role of SLA2 as a biomarker for predicting response to anti‐PD‐1 immunotherapy in STAD.

The TME plays a pivotal role in both tumour progression and immune evasion. To assess the potential influence of SLA2 expression on the TME, we employed TIMER 3.0 to evaluate its correlation with immune cell infiltration. Our analysis revealed that SLA2 expression was significantly positively correlated with the stromal scores, immune scores and ESTIMATE scores in STAD, suggesting its involvement in both stromal and immune components within the TME (Figure 3A). Moreover, SLA2 expression was positively correlated with the infiltration of CD8^+^ T cells, CD4^+^ T cells, neutrophils, myeloid dendritic cells, natural killer (NK) cells, macrophages, B cells and regulatory T cells (Tregs) (Figure 3B). Additionally, gene co‐expression analyses were performed using TISIDB to investigate the relationship between SLA2 and immune‐related genes across diverse tumour types. We systematically examined key immunological regulators such as major histocompatibility complex (MHC) genes, immunostimulatory molecules, immune checkpoint inhibitors, chemokines and chemokine receptors. The results demonstrated that SLA2 expression was positively correlated with the majority of immune cell subsets across multiple malignancies, particularly in STAD (Figure S1). Furthermore, nearly all evaluated immune‐related genes exhibited significant associations with SLA2, predominantly positive correlations (Figure S1). Collectively, these findings highlight the important regulatory role of SLA2 in modulating the immunological landscape of the TME.

We utilized the TIDE algorithm to evaluate the association between SLA2 expression and the potential clinical efficacy of immunotherapy. As shown in Figure 3C, SLA2 expression in STAD was significantly positively correlated with the dysfunction score and inversely correlated with the exclusion score. Moreover, higher SLA2 expression levels were strongly associated with increased expression of IFNG and CD274, both of which are known to promote tumour immune evasion. SLA2 expression was negatively correlated with the infiltration of myeloid‐derived suppressor cells (MDSCs) and tumour‐associated M2 macrophages, both of which are well‐established contributors to T cell exclusion within the TME. To assess SLA2's association with immunotherapy response, we analysed RNA‐seq data from the PRJEB25780 cohort (*n* = 45, advanced gastric cancer patients treated with pembrolizumab monotherapy) using TIMER3. SLA2 transcript abundance was significantly higher in responders (CR/PR) than in non‐responders (SD/PD) (Figure 3D). Furthermore, ROC curve analysis was employed to evaluate the role of SLA2 expression as a biomarker for predicting response to anti‐PD‐1 immunotherapy in GC. SLA2 expression has modest yet significant predictive value for anti‐PD‐1 immunotherapy response in GC (AUC = 0.661; Figure 3E). Taken together, these data indicate that SLA2 is associated with the TME and immune evasion in GC.

### 3.4 SLA2 Is Mainly Expressed in CD8 ^+^ T Cells in GC TME

**FIGURE 4:**
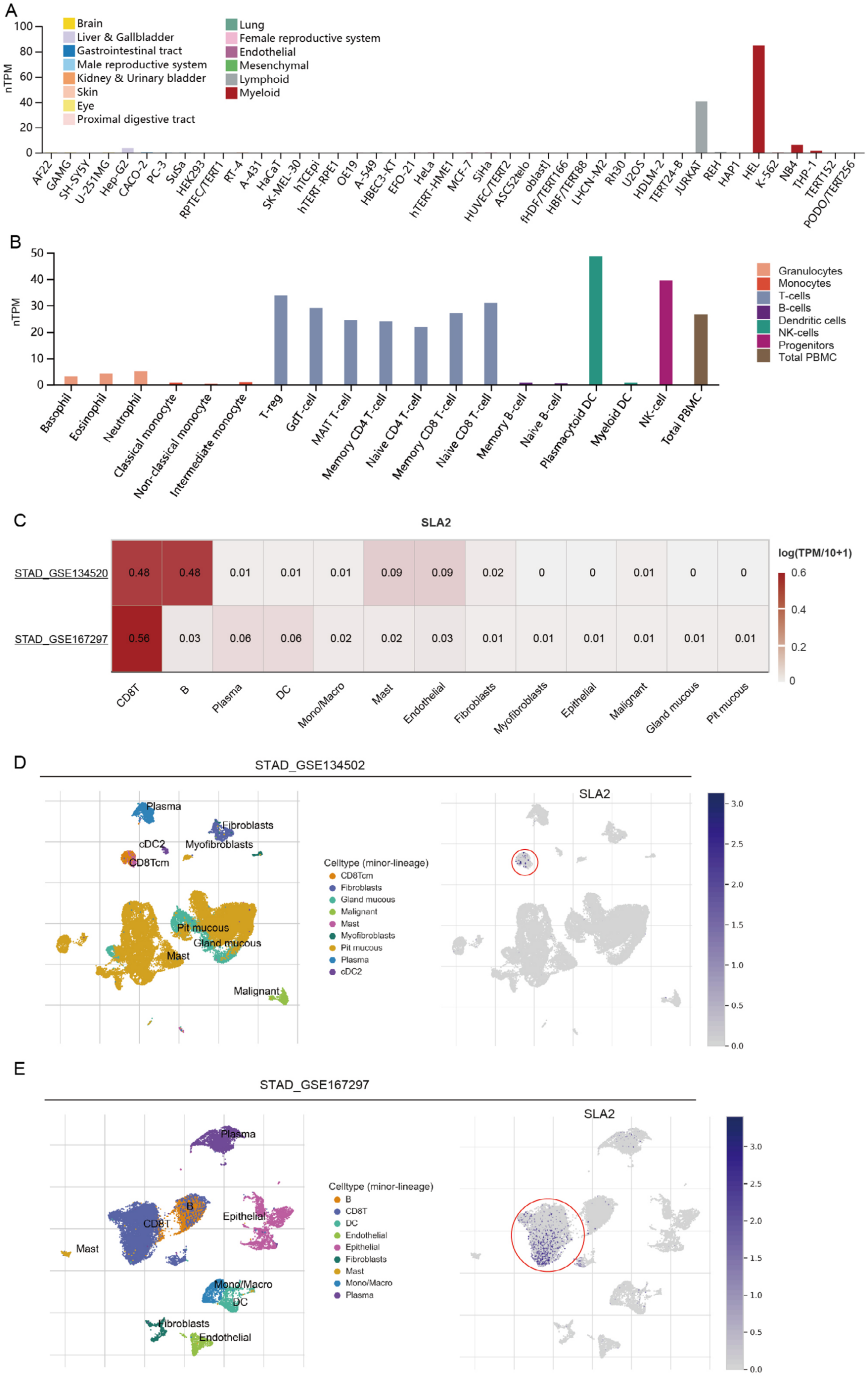
*SLA2 is enriched in CD8* ^+^ *T cells within the GC TME*. (A) Expression profile of SLA2 across a collection of widely used cell lines from diverse tissue origins based on the HPA database. (B) SLA2 expression levels in various immune cell types sourced from the HPA database. (C) SLA2 expression patterns in TME‐associated cell populations from two independent single‐cell RNA‐seq datasets of STAD. (D–E) Visualization of SLA2 distribution across distinct cell subpopulations in the STAD_GSE134502 and STAD_GSE167297 datasets obtained from the TISCH database.

Utilizing data from the HPA database, we systematically evaluated SLA2 expression across a panel of well‐established cell lines derived from diverse tissue sources. Our analysis demonstrated that SLA2 is predominantly expressed in lymphoid‐lineage cells (JURKAT) and myeloid‐lineage cells (HEL, NEB4 and THP‐1) (Figure 4A). A more detailed examination of immune cell subpopulations revealed high SLA2 expression in plasmacytoid dendritic cells (pDCs), natural killer (NK) cells, regulatory T cells (Tregs) and various T cell subsets (Figure 4B). To precisely identify SLA2‐expressing cell subsets within the GC TME, we performed single‐cell RNA sequencing analysis using two independent STAD datasets (STAD_GSE134502 and STAD_GSE167297) (Figure 4C). Among all major cell types analysed, SLA2 is predominantly expressed in CD8^+^ T cells in both datasets (Figure 4D,E). Collectively, these results suggest that SLA2 may play a role in shaping the immunological architecture of the GC TME.

### 3.5 SLA2 Promotes Exhaustion in CD8 ^+^ T Cells

**FIGURE 5:**
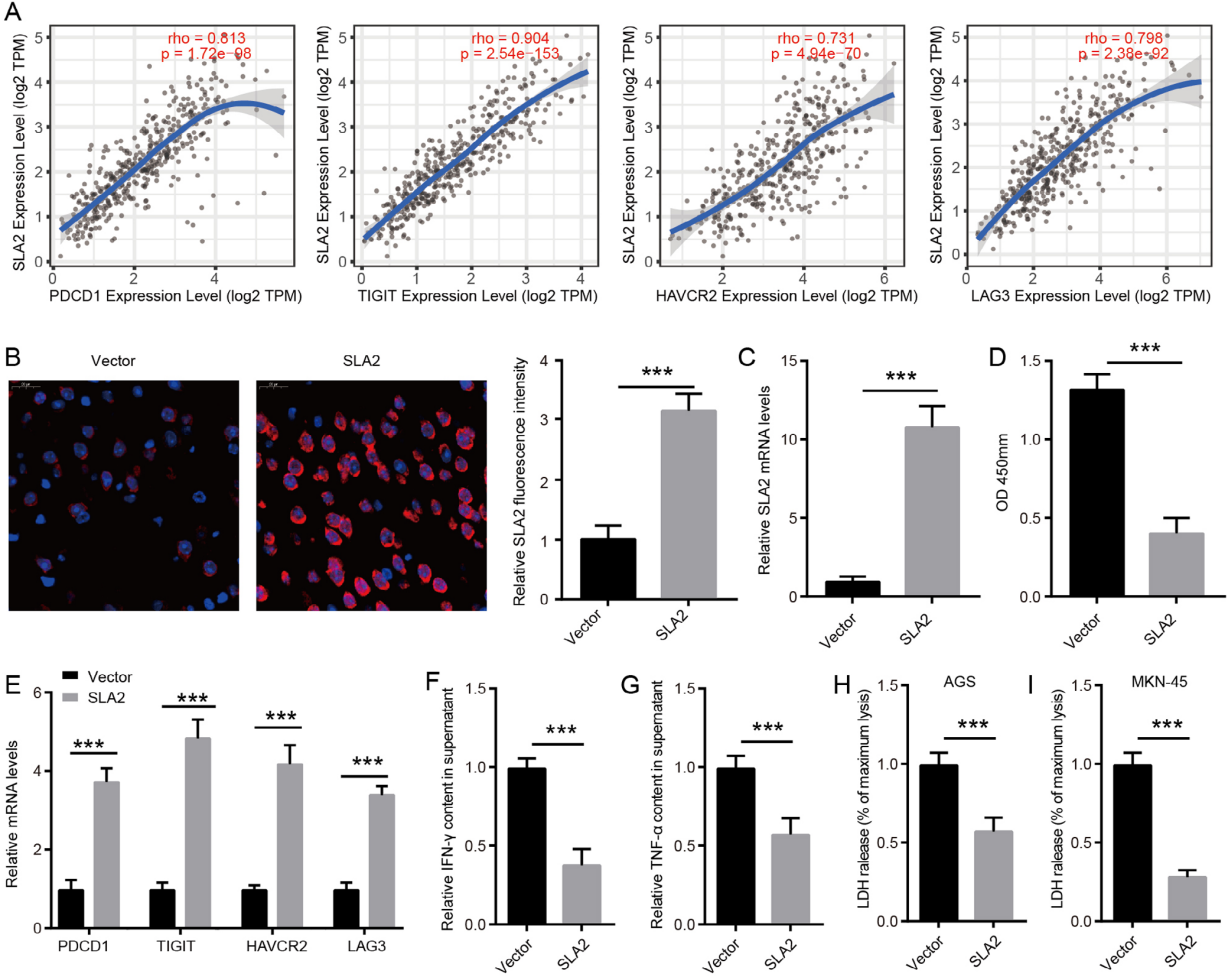
SLA2 overexpression enhances the exhaustion of CD8^+^ *T cells*. (A) Correlation between SLA2 expression and the expression of exhaustion markers PDCD1, TIGIT, HAVCR2 and LAG3 in STAD. (B) Immunofluorescence staining was employed to assess SLA2 protein expression in CD8^+^ T cells (*n* = 3). (C) SLA2 mRNA expression in CD8^+^ T cells was quantified by qRT‐PCR (*n* = 3). (D) CD8^+^ T cell proliferation was evaluated using the CCK‐8 assay (*n* = 3). (E) mRNA expression levels of PDCD1, TIGIT, HAVCR2 and LAG3 in CD8^+^ T cells were determined by qRT‐PCR (*n* = 3). (F–G) IFN‐γ and TNF‐α concentrations in CD8^+^ T cell culture supernatants were measured by ELISA (*n* = 3). (H–I) LDH release into the cell supernatant was assessed following cytotoxicity assays (*n* = 3). Statistical significance was assessed by unpaired Student's t‐test in Figure 5B–I: ***p < 0.001.

The previous findings demonstrate that SLA2 is highly enriched in CD8^+^ T cells within the GC TME and its function as a direct negative regulator of TCR signalling positions it as a mechanistically plausible contributor to T cell exhaustion, a state strongly associated with immunotherapeutic efficacy. Building on this foundation, we directly interrogated the functional role of SLA2 in driving or sustaining CD8^+^ T cell exhaustion. We found that SLA2 expression in STAD was significantly correlated with the expression of established markers of CD8^+^ T cell exhaustion, including PDCD1, TIGIT, HAVCR2 and LAG3 (Figure 5A). To further explore the functional role of SLA2 in CD8^+^ T cell exhaustion, we overexpressed SLA2 in activated CD8^+^ T cells. SLA2 overexpression markedly upregulated both protein and mRNA levels in CD8^+^ T cells (Figure 5B,C). A CCK‐8 assay indicated that SLA2 overexpression inhibited CD8^+^ T cell proliferation (Figure 5D). Furthermore, SLA2 overexpression increased the mRNA expression of PDCD1, TIGIT, HAVCR2 and LAG3 (Figure 5E) and reduced the secretion of IFN‐γ and TNF‐α (Figure 5F,G). An LDH‐based cytolysis assay revealed that SLA2 overexpression significantly impaired T cell cytotoxic function (Figure 5H,I). Collectively, these results suggest that SLA2 may play a role in modulating CD8^+^ T cell exhaustion.

To further verify SLA2's functional contribution, we carried out loss‐of‐function experiments using shRNAs to achieve efficient and specific knockdown of SLA2 expression in in vivo‐generated exhausted CD8 T cells. We developed an in vitro human CD8^+^ T cell exhaustion model by applying repeated stimulation with soluble anti‐CD3 and anti‐CD28 antibodies. This system faithfully reproduces key features of exhaustion, such as upregulation of inhibitory receptors (PDCD1, TIGIT, HAVCR2 and LAG3) and diminished production of TNF‐α and IFN‐γ, along with reduced cytotoxic activity against GC cells (Figure S2). We found that the SLA2 mRNA level was increased in in vivo‐generated exhausted CD8^+^ T cells (Figure S2A). Knockdown of SLA2 by its shRNAs significantly reduced SLA2 mRNA levels in exhausted CD8^+^ T cells (Figure S2A). Moreover, SLA2 knockdown significantly downregulated exhaustion‐associated surface markers (Figure S2B–E), restored effector function, as evidenced by increased TNF‐α and IFN‐γ production (Figure S2F–G), and enhanced cytotoxic activity against GC cells (Figure S2H–I). Collectively, these findings demonstrate that SLA2 suppression reverses key functional impairments in exhausted CD8^+^ T cells.

### 3.6 Methylation Data Analysis of SLA2 in STAD

**FIGURE 6:**
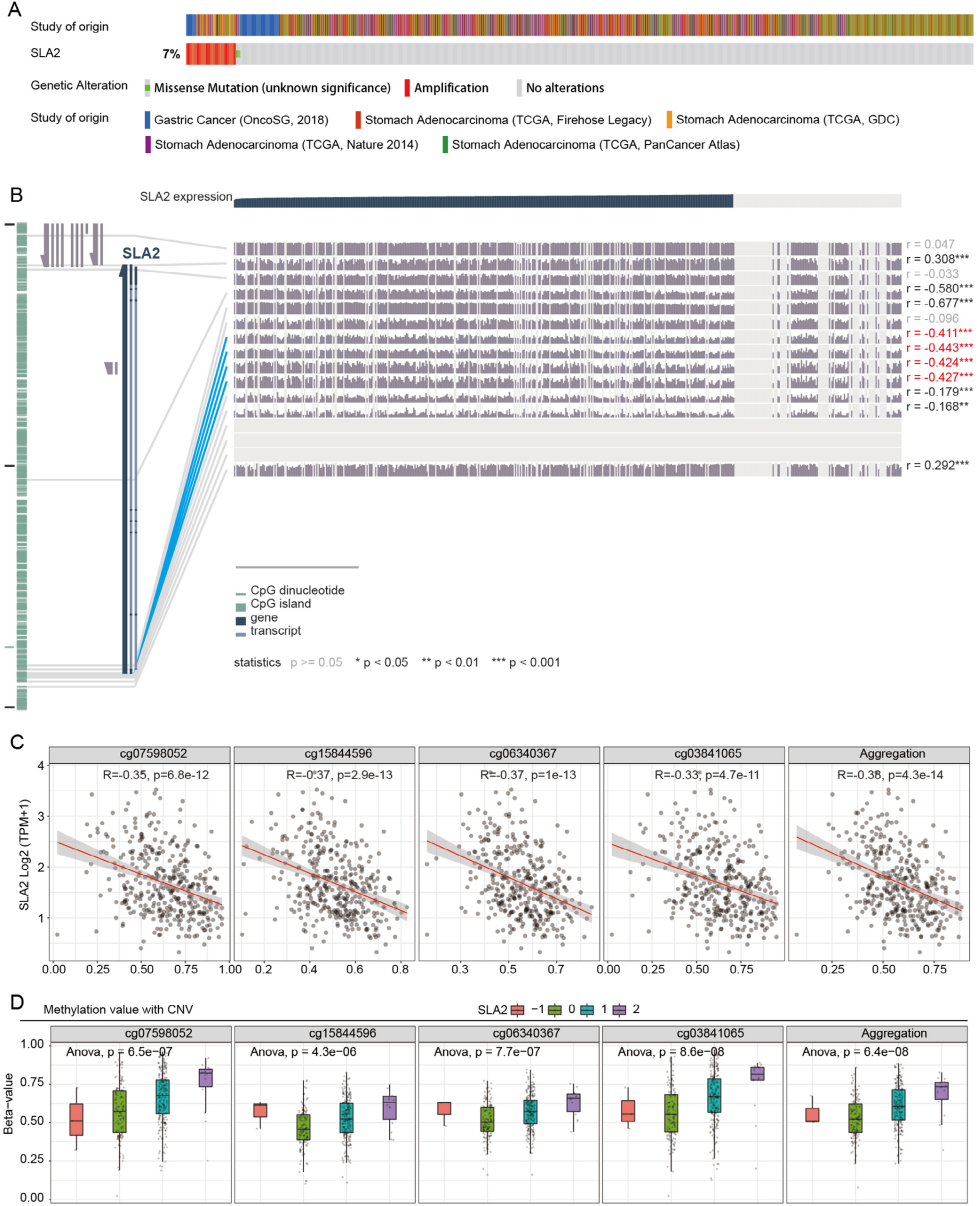
*Genetic alterations and DNA methylation changes of SLA2 in STAD*. (A) Mutation frequency of SLA2 in STAD derived from the cBioPortal database. (B) Visualization of the SLA2 DNA sequence showing methylation sites in relation to gene expression in STAD using the MEXPRESS tool. (C) Correlation between promoter CpG island methylation levels and SLA2 expression in STAD, analysed via the SMART. (D) Methylation levels at CpG sites within the SLA2 promoter region stratified by CNV status.

Next, we sought to investigate the regulatory mechanisms underlying SLA2 upregulation in gastric cancer. Genetic alterations of SLA2 in GC were analysed using data from the cBioPortal database. The mutation frequency of SLA2 in GC was approximately 7%, with ‘amplification’ identified as the predominant form of copy number alteration (CNA) (Figure 6A). Patients harbouring CNA amplification exhibited higher levels of SLA2 expression, suggesting a potential link between genomic copy changes and transcript abundance. Beyond CNA, gene expression is subject to epigenetic regulation, particularly through DNA methylation. To evaluate the methylation status of SLA2 in STAD, we performed methylation analysis using the MEXPRESS platform. Multiple CpG islands were identified within the SLA2 promoter region, and their methylation levels were significantly inversely correlated with SLA2 expression (Figure 6B). This inverse relationship between promoter CpG methylation and SLA2 expression was further confirmed using the SMART platform, which showed that methylation at specific CpG sites in the SLA2 promoter—cg07598052, cg15844596, cg06340367 and cg03841065—was negatively correlated to SLA2 expression (Figure 6C). Notably, copy number variations (CNVs) may also influence DNA methylation patterns; our results indicated a significant association between methylation levels at these CpG sites and CNV status (Figure 6D). Collectively, these findings support that DNA methylation may serve as a potential regulatory mechanism for modulating SLA2 expression in GC.

### 3.7 Histone Modifications May Influence SLA2 Expression in T Cells

**FIGURE 7:**
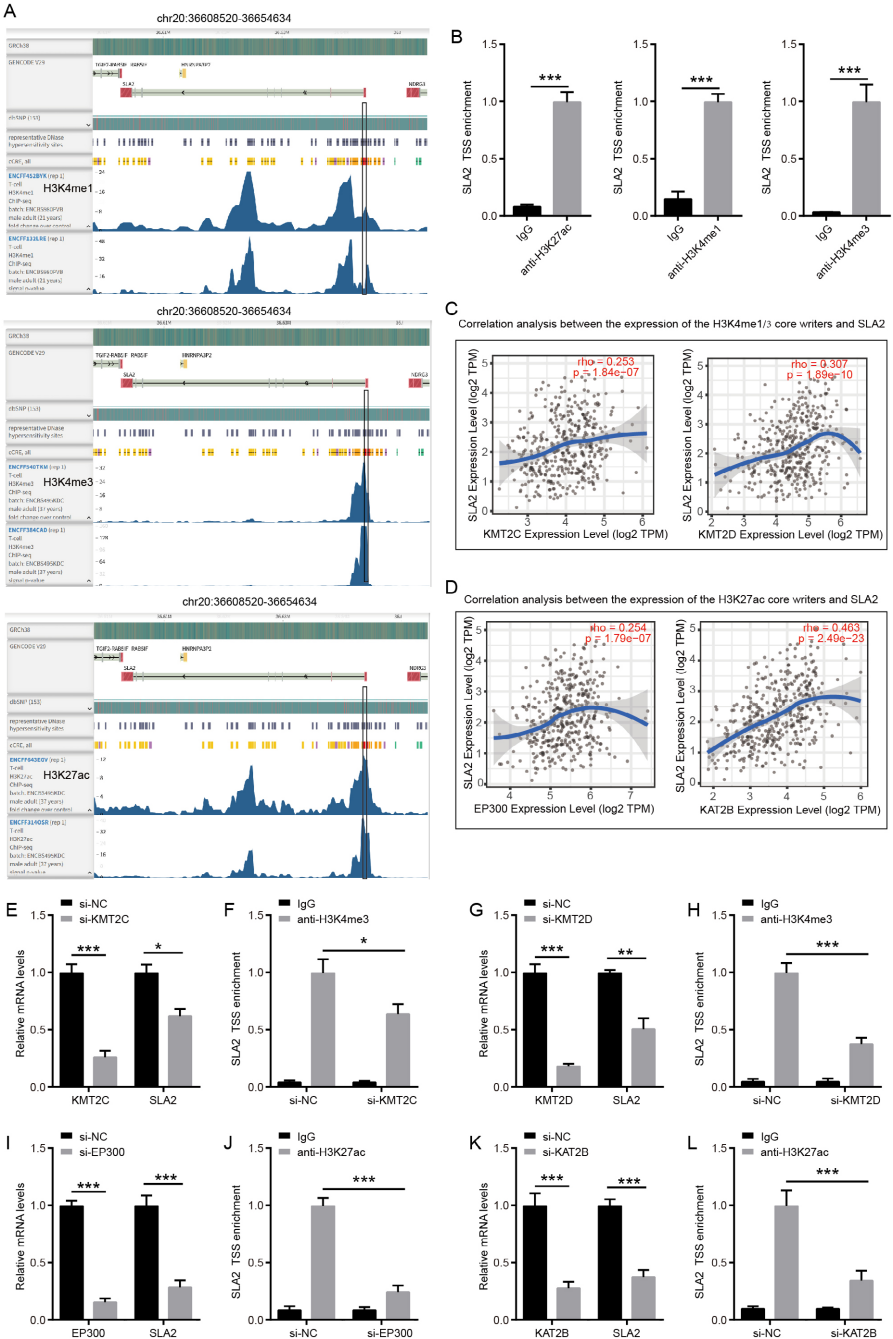
*Histone modifications are involved in the regulation of SLA2 activation in CD8* ^+^ *T cells*. (A) ChIP‐seq data from the ENCODE database showed enrichment of H3K4me1, H3K4me3 and H3K27ac at the SLA2 locus in T cells. (B) ChIP assays were conducted to detect the enrichment of H3K4me1, H3K4me3 and H3K27ac at the TSS of the SLA2 gene in CD8^+^ T cells (*n* = 3). Statistical significance was assessed by unpaired Student's t‐test: ***p < 0.001. (C) Correlation between SLA2 expression and that of the core H3K4me1/3 methyltransferases KMT2C and KMT2D in STAD. (D) Correlation between SLA2 expression and that of the core H3K27ac acetyltransferases EP300 and KAT2B in STAD. (E) mRNA expression levels of KMT2C and SLA2 in CD8^+^ T cells with or without KMT2C knockdown were determined by qRT‐PCR (*n* = 3). (F) ChIP assays were conducted to detect the enrichment of H3K4me at the TSS of the SLA2 gene in CD8^+^ T cells with or without KMT2C knockdown (*n* = 3). (G) mRNA expression levels of KMT2D and SLA2 in CD8^+^ T cells with or without KMT2D knockdown were determined by qRT‐PCR (*n* = 3). (H) ChIP assays were conducted to detect the enrichment of H3K4me at the TSS of the SLA2 gene in CD8^+^ T cells with or without KMT2D knockdown (*n* = 3). (I) mRNA expression levels of EP300 and SLA2 in CD8^+^ T cells with or without EP300 knockdown were determined by qRT‐PCR (*n* = 3). (J) ChIP assays were conducted to detect the enrichment of H3K27ac at the TSS of the SLA2 gene in CD8^+^ T cells with or without EP300 knockdown (*n* = 3). (K) mRNA expression levels of KAT2B and SLA2 in CD8^+^ T cells with or without KAT2B knockdown were determined by qRT‐PCR (*n* = 3). (L) ChIP assays were conducted to detect the enrichment of H3K27ac at the TSS of the SLA2 gene in CD8^+^ T cells with or without KAT2B knockdown (*n* = 3). Statistical significance was assessed by unpaired Student's t‐test in Figure 7E–L: *p < 0.05, **p < 0.01, ***p < 0.001.

Histone modifications at gene promoters critically regulate downstream gene expression by modulating chromatin conformation and thereby controlling transcription factor access to DNA. Histone H3 lysine 4 trimethylation (H3K4me3) marks active promoters located at transcription start sites (TSS), whereas mono‐methylation of H3K4 (H3K4me1) and acetylation of histone H3 lysine 27 (H3K27ac) characterize enhancer regions that facilitate gene activation [20, 21]. To map these histone modifications in T cells, chromatin immunoprecipitation followed by sequencing (ChIP‐seq) data from the ENCODE platform was analysed. The results revealed pronounced enrichment of H3K4me3 and H3K27ac near the TSS, indicative of active promoter states, while regions upstream and within the gene body displayed signatures of enhancer activity, including H3K4me1 and H3K27ac (Figure 7A). Furthermore, ChIP assays detected robust peaks of H3K4me1, H3K4me3 and H3K27ac across the TSS region of the SLA2 gene in CD8^+^ T cells (Figure 7B). Moreover, in STAD, SLA2 expression showed a significant positive correlation with the expression levels of key enzymes responsible for H3K4 methylation (the methyltransferases KMT2C and KMT2D) (Figure 7C) as well as those mediating H3K27 acetylation (EP300 and KAT2B) (Figure 7D). Moreover, shRNA‐mediated knockdown of KMT2C significantly reduced SLA2 mRNA levels and concomitantly diminished H3K4me3 enrichment at the SLA2 TSS (Figure 7E,F). Similarly, KMT2D knockdown recapitulated these effects—reducing both SLA2 transcript abundance and TSS‐associated H3K4me3 (Figure 7G,H). These data indicated a direct link between this histone methyltransferase and SLA2 transcription activation. Besides, knockdown of the histone acetyltransferases EP300 or KAT2B also led to significant decreases in SLA2 mRNA and H3K27ac occupancy at the SLA2 TSS (Figure 7I–L), indicating a direct link between this histone acetyltransferase and SLA2 transcription activation. These findings suggest that epigenetic regulation through histone modification may modulate SLA2 transcription.

## 4 Discussion

There is an urgent requirement to identify new prognostic biomarkers and potential therapeutic targets for GC. In this study, we found that SLA2 was elevated in GC tissues and was associated with poor prognosis. SLA2 may modulate the TME and promote immune evasion by affecting CD8^+^ T cell exhaustion in GC, suggesting that SLA2 may represent a promising immune checkpoint–like target for cancer immunotherapy.

In this study, we investigated the potential role of SLA2 in CD8^+^ T cell exhaustion. We selected this biological context for three interrelated reasons. First, spatial expression specificity: single‐cell transcriptomic profiling of the GC TME revealed that SLA2 expression is significantly enriched in the CD8^+^ T cell compartment relative to other immune subsets. Second, mechanistic plausibility: as a well‐established negative regulator of TCR signalling, SLA2 intrinsically modulates the activation, effector function and functional persistence of CD8^+^ T cells; under conditions of chronic antigen exposure—such as those prevailing in solid tumours—sustained SLA2 upregulation is therefore predicted to impair TCR signal transduction and accelerate the transition to an exhausted state. Third, translational significance: given that the functional integrity of tumour‐infiltrating CD8^+^ T cells is a critical determinant of response to immune checkpoint blockade, elucidating SLA2‐mediated regulation of exhaustion not only provides a mechanistic basis for the observed clinical associations—including its correlation with immune evasion and diminished therapeutic efficacy—but also establishes a rational foundation for exploring SLA2‐targeted interventions in combination immunotherapies.

Notably, the role of SLA2 is context‐dependent and appears to be more nuanced. While its overexpression inhibits tumour formation in acute myeloid leukaemia [22], SLA2 has also been implicated as a critical regulator of malignant progression in non‐small cell lung cancer and head and neck squamous cell carcinoma [16, 17]. Adding to this complexity, we found that elevated SLA2 expression is associated with poor prognosis in GC patients and promotes immune evasion, notably by enhancing CD8^+^ T cell exhaustion. Taken together, this evidence underscores that SLA2's function is not uniform but exhibits strong tissue‐ and tumour‐type specificity. The TME is a complex ecosystem in which SLA2 likely exerts distinct roles across cell types. This complexity positions SLA2 as a context‐dependent signalling node, and future investigations must dissect its compartment‐specific mechanisms to fully elucidate its role in GC pathogenesis and therapeutic potential.

A seemingly paradoxical observation emerged from this study: high SLA2 expression correlated with an elevated TIDE dysfunction score—indicative of immune evasion—yet was concurrently associated with a favourable predicted response to immunotherapy. We posit that this apparent paradox reflects the biologically coherent interplay between CD8^+^ T cell infiltration and functional exhaustion within the GC TME, rather than a genuine inconsistency. Notably, this pattern mirrors the well‐documented dual role of canonical immune checkpoint molecules, including PD‐1 and LAG‐3. Robust clinical evidence demonstrates that intratumoral overexpression of PD‐1 or LAG‐3 serves both as a definitive marker of T cell exhaustion and as one of the strongest positive predictors of clinical response to ICIs [23, 24, 25]. Critically, effective checkpoint inhibition requires a pre‐existing pool of tumour‐infiltrating, antigen‐experienced T cells—even if exhausted—as these cells constitute the essential cellular substrate for therapeutic reinvigoration. Moreover, the TIDE dysfunction score is derived from transcriptomic signatures reflecting both CD8^+^ T cell infiltration levels and exhaustion‐associated gene expression. Our data demonstrate that SLA2 is robustly expressed in tumour‐infiltrating CD8^+^ T cells within the GC microenvironment and exhibits strong positive correlations with canonical exhaustion markers—including PDCD1, TIGIT, HAVCR2 and LAG3—as well as with functional impairments such as reduced cytokine production and cytolytic activity. Analogous to PD‐1 and LAG‐3, elevated SLA2 expression thus identifies tumours enriched for exhausted yet phenotypically plastic CD8^+^ T cells. Consequently, high SLA2 expression drives classification as ‘high dysfunction’ by TIDE, capturing the current state of immunosuppression; however, its association with improved immunotherapy response reflects the latent functional reversibility of these cells upon checkpoint blockade. Importantly, TIDE—a transcriptome‐based computational tool—does not explicitly model the dynamic reversibility of exhaustion states. Instead, high SLA2 expression co‐occurs with substantial CD8^+^ T cell infiltration, defining an ‘inflamed’ or ‘immune‐hot’ tumour phenotype—a prerequisite for immunotherapy responsiveness. This ‘exhausted but reversible’ state represents a therapeutically actionable condition, and the consistent co‐expression of SLA2 with PD‐1 and LAG‐3 further validates its utility as a biomarker of this clinically relevant phenotype. In summary, the dual association of high SLA2 expression with immune evasion and favourable immunotherapy outcomes is not contradictory; it faithfully captures a TME characterized by abundant, infiltrating CD8^+^ T cells undergoing reversible exhaustion. SLA2 therefore emerges as a promising predictive biomarker for identifying GC patients who—despite exhibiting molecular signatures of immune escape—retain the cellular prerequisites for clinical benefit from immune checkpoint blockade.

DNA methylation and histone modification usually work together to regulate gene expression. Within the TME, CD8^+^ T cells frequently become exhausted, a state linked to epigenetic changes. These exhausted T cells display distinct patterns of DNA methylation and histone modifications, which function as molecular ‘locks’ that maintain them in a less functional state [26, 27, 28]. Therefore, reversing the epigenetic programming of T cells is a cutting‐edge direction in current immunotherapy research. In this study, we explored the underlying mechanism of increased SLA2 expression in GC and found that SLA2 overexpression was inversely associated with the methylation levels at CpG sites cg07598052, cg15844596, cg06340367 and cg03841065, located within the promoter region of the SLA2 gene. These results suggest a potential regulatory link between promoter methylation and SLA2 expression. However, we identified a potential regulatory link between promoter methylation and SLA2 expression using bioinformatics without wet‐lab validation. This will require bisulfite conversion of genomic DNA extracted from sorted CD8^+^ T‐cell subsets, followed by targeted sequencing of the SLA2 promoter region to quantitatively compare methylation levels between functionally exhausted and non‐exhausted populations.

Moreover, increased levels of H3K4me1, H3K4me3 and H3K27ac were observed around the TSS of the SLA2 gene in CD8^+^ T cells, suggesting that histone modifications may also play a role in regulating SLA2 expression. KMT2C and KMT2D, which are responsible for H3K4 methylation, regulate several signalling pathways [29, 30]. In our study, SLA2 expression in GC was found to be positively correlated with the expression levels of both KMT2C and KMT2D. The knockdown of KMT2C or KMT2D reduced SLA2 mRNA levels and concomitantly diminished H3K4me3 enrichment at the SLA2 TSS. Additionally, EP300 catalyses H3K27 acetylation at gene promoters, enhancers and super‐enhancers, leading to transcriptional activation [31, 32]. Similarly, KAT2B facilitates gene transcription by promoting H3K27ac enrichment at target promoters [33, 34]. We also observed a positive association between SLA2 expression and the levels of EP300 or KAT2B in GC. The knockdown of EP300 or KAT2B also reduced SLA2 mRNA levels and concomitantly diminished H3K27ac enrichment at the SLA2 TSS. Notably, KMT2C and KMT2D form similar multi‐subunit complexes that mediate H3K4 mono‐methylation at enhancer regions, and in coordination with EP300‐driven H3K27 acetylation, help establish an active enhancer environment conducive to long‐range activation of target genes [35]. Together, these findings suggest that epigenetic regulation through histone modification may contribute to the dysregulated expression of SLA2.

## 5 Conclusions

In summary, our findings indicate that SLA2 may serve as a potential biomarker for poor prognosis and immune evasion in GC. SLA2 may modulate the TME and promote immune evasion by enhancing CD8^+^ T cell exhaustion. These results contribute to a deeper understanding of SLA2's involvement in tumour development and provide a basis for future investigations.

## Author Contributions

**Xiaoyan Li:** software, investigation, visualization, formal analysis. **Rongchao Xiang:** resources, supervision, writing – review and editing. **Yujia Zhang:** conceptualization, data curation, methodology, investigation, visualization, formal analysis, writing – original draft, writing – review and editing.

## Consent

All authors have read and agreed to publish this manuscript.

## Conflicts of Interest

The authors declare no conflicts of interest.

## Supporting information


**Figure S1:** Association of SLA2 and immunoregulators. Association of SLA2 expression with immune cell populations (A), immunostimulatory molecules (B), immune checkpoint inhibitors (C), MHC molecules (D), chemokines (E) and chemokine receptors (F). Positive correlations are represented in red, and negative correlations are shown in blue.
**Figure S2:** SLA2 knockdown improved the function of exhausted CD8 T cells. (A) SLA2 mRNA expression in CD8^+^ T cells with indicated treatments was quantified by qRT‐PCR (*n* = 3). (B–E) The mRNA expression levels of PDCD1, TIGIT, HAVCR2 and LAG3 in CD8^+^ T cells with indicated treatments were determined by qRT‐PCR (*n* = 3). (F–G) IFN‐γ and TNF‐α concentrations in CD8^+^ T cell culture supernatants were measured by ELISA (*n* = 3). (H–I) LDH release into the cell supernatant was assessed following cytotoxicity assays (*n* = 3). Statistical significance was assessed by one‐way ANOVA followed by Tukey's post hoc test for multiple comparisons: *p < 0.05, **p < 0.01, ***p < 0.001.

## Data Availability

The data that support the findings of this study are available from the corresponding author upon reasonable request.
