# Supplementary material for: SLA2 is Associated With Immune evasion and Exhaustion of CD8 + T Cells in Gastric Cancer
Source: J Cell Mol Med. 2026 May 10;30(9):e71164. doi: 10.1111/jcmm.71164 (PMC13158163; doi:10.1111/jcmm.71164)
Supplement: Supplementary file 1 — Figure S1: Association of SLA2 and immunoregulators. Association of SLA2 expression with immune cell populations (A), immunostimulatory molecules (B), immune checkpoint inhibitors (C), MHC molecules (D), chemokines (E) and chemokine receptors (F). Positive correlations are represented in red, and negative correlations are shown in blue. Figure S2: SLA2 knockdown improved the function of exhausted CD8 T cells. (A) SLA2 mRNA expression in CD8+ T cells with indicated treatments was quantified by qRT‐PCR (n = 3). (B–E) The mRNA expression levels of PDCD1, TIGIT, HAVCR2 and LAG3 in CD8+ T cells with indicated treatments were determined by qRT‐PCR (n = 3). (F–G) IFN‐γ and TNF‐α concentrations in CD8+ T cell culture supernatants were measured by ELISA (n = 3). (H–I) LDH release into the cell supernatant was assessed following cytotoxicity assays (n = 3). Statistical significance was assessed by one‐way ANOVA followed by Tukey's post hoc test for multiple comparisons: *p < 0.05, **p < 0.01, ***p < 0.001. [file JCMM-30-e71164-s001.docx]

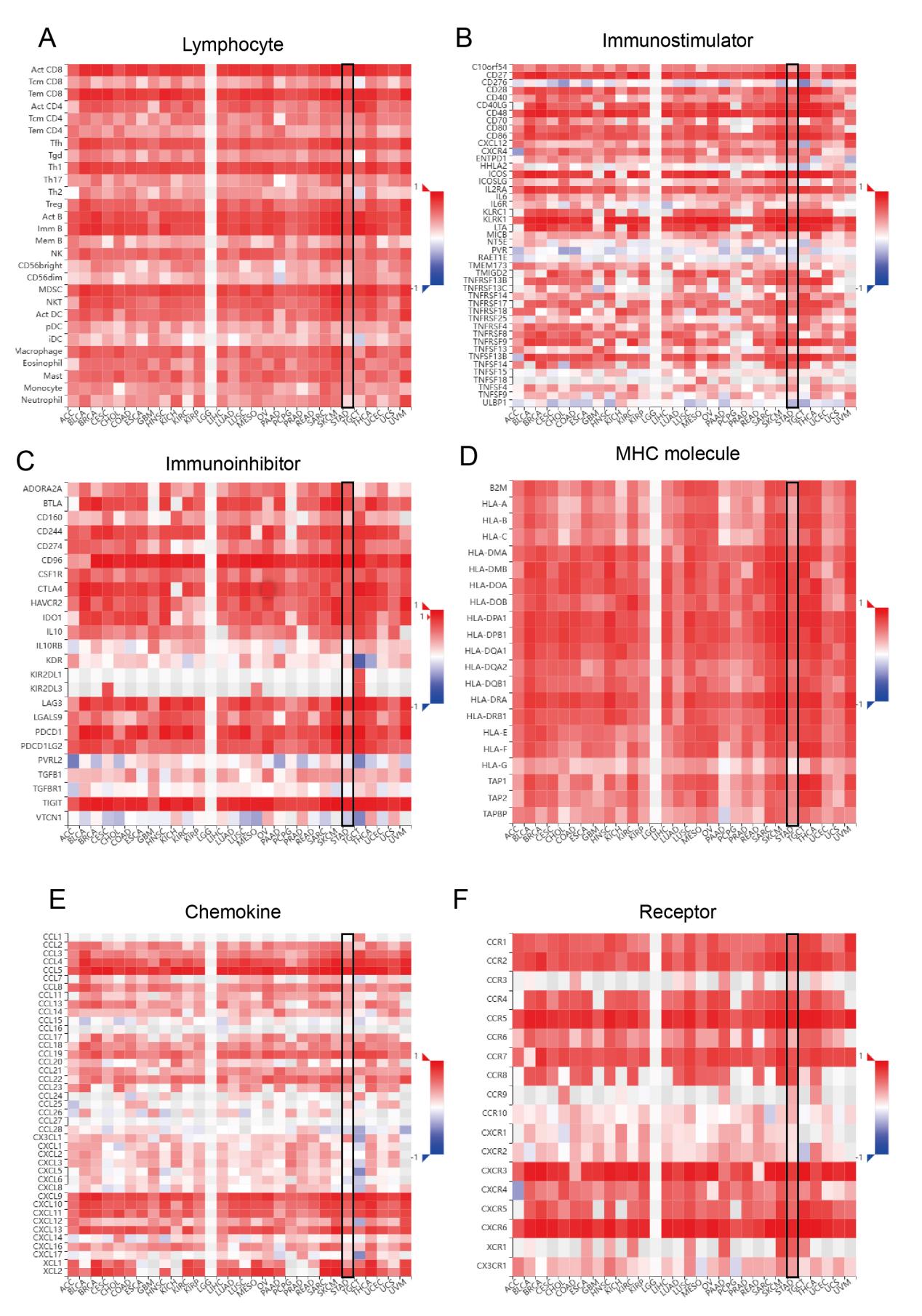


**Figure S1 Association of SLA2 and immunoregulators.** Association of SLA2 expression with immune cell populations (A), immunostimulatory molecules (B), immune checkpoint inhibitors (C), MHC molecules (D), chemokines (E), and chemokine receptors (F). Positive correlations are represented in red, and negative correlations are shown in blue.


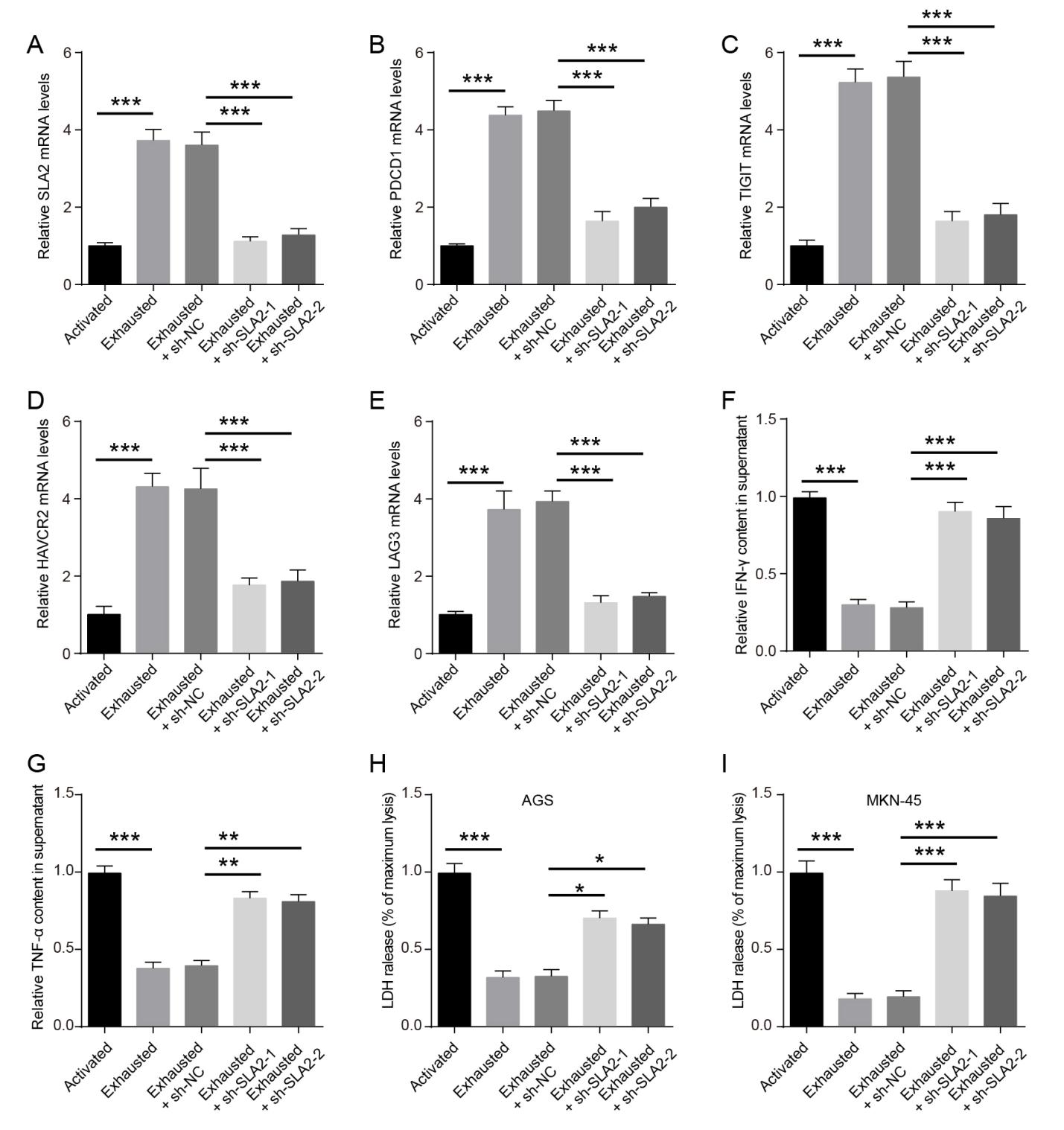


**Figure S2 SLA2 knockdown improved the function of exhausted CD8 T cells.** (A) SLA2 mRNA expression in CD8⁺ T cells with indicated treatments was quantified by qRT-PCR (n = 3). (B-E) The mRNA expression levels of PDCD1, TIGIT, HAVCR2, and LAG3 in CD8⁺ T cells with indicated treatments were determined by qRT-PCR (n = 3). (F–G) IFN-γ and TNF-α concentrations in CD8⁺ T cell culture supernatants were measured by ELISA (n = 3). (H-I) LDH release into the cell supernatant was assessed following cytotoxicity assays (n = 3). Statistical significance was assessed by one-way ANOVA followed by Tukey’s post hoc test for multiple comparisons: *P < 0.05, **P < 0.01, ***P < 0.001.
